# Role of Functionalized Peptides in Nanomedicine for Effective Cancer Therapy

**DOI:** 10.3390/biomedicines12010202

**Published:** 2024-01-16

**Authors:** Kibeom Kim, Myoung-Hwan Park

**Affiliations:** 1 Convergence Research Center, Nanobiomaterials Institute, Sahmyook University, Seoul 01795, Republic of Korea; mpark@syu.ac.kr; 2 Department of Chemistry and Life Science, Sahmyook University, Seoul 01795, Republic of Korea; 3 Department of Convergence Science, Sahmyook University, Seoul 01795, Republic of Korea

**Keywords:** cancer therapy, functionalization, nanomedicine, nanoparticle, peptide

## Abstract

Peptide-functionalized nanomedicine, which addresses the challenges of specificity and efficacy in drug delivery, is emerging as a pivotal approach for cancer therapy. Globally, cancer remains a leading cause of mortality, and conventional treatments, such as chemotherapy, often lack precision and cause adverse effects. The integration of peptides into nanomedicine offers a promising solution for enhancing the targeting and delivery of therapeutic agents. This review focuses on the three primary applications of peptides: cancer cell-targeting ligands, building blocks for self-assembling nanostructures, and elements of stimuli-responsive systems. Nanoparticles modified with peptides improved targeting of cancer cells, minimized damage to healthy tissues, and optimized drug delivery. The versatility of self-assembled peptide structures makes them an innovative vehicle for drug delivery by leveraging their biocompatibility and diverse nanoarchitectures. In particular, the mechanism of cell death induced by self-assembled structures offers a novel approach to cancer therapy. In addition, peptides in stimuli-responsive systems enable precise drug release in response to specific conditions in the tumor microenvironment. The use of peptides in nanomedicine not only augments the efficacy and safety of cancer treatments but also suggests new research directions. In this review, we introduce systems and functionalization methods using peptides or peptide-modified nanoparticles to overcome challenges in the treatment of specific cancers, including breast cancer, lung cancer, colon cancer, prostate cancer, pancreatic cancer, liver cancer, skin cancer, glioma, osteosarcoma, and cervical cancer.

## 1. Introduction

Cancer is a leading cause of death worldwide, accounting for approximately 10 million deaths in 2020 [1,2]. Cancer treatments include chemotherapy, phototherapy, radiation therapy, and surgery [3,4,5,6,7,8,9,10,11,12]. Chemotherapy is a traditional treatment for cancer and can effectively treat cancer, even at low doses [13,14,15]. However, since many cancer drugs are hydrophobic, injecting the appropriate drug concentration required for treatment is difficult, and direct drug injection often causes side effects owing to non-specific effects on cancer and normal cells [16,17,18,19]. Additionally, the injected drugs are removed by the reticuloendothelial system (RES), and only a small fraction of the drug is delivered to the cancer cells, making it difficult to reach a sufficient concentration for treatment [20,21,22,23,24]. Drug delivery systems have been developed over the past few years [25,26,27]. Mesoporous silica nanoparticles (MSNP), gold nanoparticles (GNP), quantum dots (QD), polymeric nanoparticles, and metal-organic nanoparticle frameworks (MOF) have received attention as nanomedicine platforms because they accumulate in cancer tissues owing to the enhanced permeation retention (EPR), which not only helps selective drug delivery but also allows porous particles to load the cancer drugs in their pores without specific modification [23,28,29,30,31,32,33,34,35,36]. However, these nanomaterial-based systems also have problems with biocompatibility, RES escape, controlled release of loaded drugs, and selective cancer cell targeting [37,38,39,40,41,42]. Therefore, many researchers have modified various nanomedicine substances to add new functions to cancer treatment [43,44,45,46,47].

A peptide is a short chain of amino acids, less than 50 amino acids, with two or more amino acids linked through a peptide bond [48]. Peptides provide a cancer-targeting ability to nanomedicine, reducing drug side effects and increasing treatment effectiveness. In addition, the peptide itself self-assembles and is not only used as a drug carrier that carries drugs and safely delivers them to cancer cells but also treats cancer with its self-assembly structure. Lastly, peptides facilitate the controlled drug release of nanomedicine by responding to the stimuli [49,50,51,52,53]. In this paper, we introduce a system using peptides or peptide-modified nanoparticles to overcome the challenges in the treatment of specific cancer (breast, lung, colon, prostate, pancreatic, liver, skin, glioma, osteosarcoma, and cervical cancer) and describe the peptide functionalization method used. One peptide can perform various functions in the nanomedicine, but in this review, one representative function of the peptide used in the study was used and each was classified into cancer-targeting ligand, the building block of the self-assembly structure, and stimuli-responding system.

## 2. Functions of Peptides

### 2.1. Cancer-Targeting Ligand

Nonspecific nanomedicine accumulation poses significant challenges in cancer treatment as they can inadvertently lead to adverse side effects by accumulating in healthy cells [54,55]. Furthermore, the cell membrane acts as a biological barrier, impeding efficient drug delivery to cancer cells [56]. The barrier function of the cell membrane hinders the penetration and absorption of therapeutic agents, making it challenging to achieve the desired drug concentration in cancer cells. To address these issues, cancer-targeting or cancer-penetrating peptides have been modified on nanoparticle surfaces, which enhances nanoparticle selectivity, allowing them to specifically target cancer cells. Cancer cells possess specific surface markers that differ from those of normal cells. Peptides recognize these cancer-specific surface markers and bind to them with high affinity. Additionally, peptides are structurally flexible, allowing them to adapt to various forms of cancer cell surface markers. This structural flexibility enables peptides to effectively bind in the complex environment of the cancer cell surface [57,58]. This targeted approach significantly improves the efficiency of drug delivery, maximizes the therapeutic effects, and minimizes potential side effects [59,60].

#### 2.1.1. Receptor-Mediated Peptide

The receptor-mediated peptide has a strong affinity with the receptor present in cancer cells, allowing it to selectively target and treat cancer. Craciun et al. developed a cancer cell-targeting system by modifying decapeptides in a gold nanoparticle-based gene delivery system [61]. Polyethylenimine (PEI), which has beta-cyclodextrin (β-CD), was modified on the gold nanoparticle surface. PEI is widely used as a versatile gene carrier with high transfection efficiency and reproducibility, both in vitro and in vivo. Moreover, it has a high positive charge owing to the amino groups in its structure and forms a polyplex via electrostatic interaction with negatively charged phosphorus, which is the DNA backbone. Therefore, it effectively loads the plasmid DNA required for gene therapy. The decapeptide (WXEAAYQRFL), which has a high affinity for MCF-7 cancer cells, endows NPs with targeting properties [62]. This system not only delivers plasmid DNA to cancer cells but also protects plasmid DNA from degradation by endonucleases. To compare the delivery efficiency between specific peptide-labeled nanoparticles (AuPEI–β-CD–Pep NPs) and unlabeled nanoparticles (AuPEI–β-CD NPs), in vitro transfection efficiency was evaluated in two cancer cell lines, HeLa and MCF-7, using pCS2+MT-Luc DNA as a reporter gene. In the result, AuPEI–β-CD–Pep NPs show higher and similar transfection efficiency than AuPEI–β-CD NPs in MCF-7 and HeLa cells, respectively. These results indicate that the peptide specifically interacted with MCF-7 cells and effectively delivered the loaded DNA. The modifications of decapeptides have enhanced the targeting capabilities of gene delivery systems based on gold nanoparticles. Moreover, systems modified with peptides showed high DNA delivery efficiency.

Zhao et al. have developed a cancer-targeting system using MOF and di-peptides [63]. MOF, a nanocarrier, is a porous material synthesized using a coordination bond between a metal ion and an organic linker and has the advantages of adjustable porosity and structure, high surface area, and the ability to load various drugs [64,65,66,67]. Zeolitic imidazolate frameworks-8 (ZIF-8) comprise zinc and dimethylimidazole and have pH-responsive biodegradation properties in addition to the characteristics of existing MOFs, thus facilitating selective drug release into the cancer cells [68]. The imidazolate linker of ZIF-8 is protonated in an acidic environment and the metal–ligand bond is broken, resulting in the collapse of the MOF. The peptide (WQPDTAHHWATL) has a strong specific affinity for prostate-specific membrane antigen (PSMA), which is highly expressed on the surface of prostate cancer cells [69]. This peptide is dimerized with a lysine residue at the COOH terminus. The dipeptide significantly enhanced the affinity and specificity of the receptor compared with the peptide monomer through the bivalent effect. The synthesized dipeptide was modified in polyethylene glycol (PEG) to increase its biocompatibility [70]. To obtain a targeted MOF drug delivery system (Di-PEG@PTX@ZIF-8), the dipeptide was modified on the surface of ZIF-8 via electrostatic interactions. Coumarin 6 (Cou6) was loaded into this system instead of paclitaxe (PTX) for studying cellular uptake. To confirm the targeting effect, Lncap, a prostate cancer cell line, was incubated with free Cou6, a peptide-free MOF drug delivery system (mPEG@Cou@ZIF-8), and Di-PEG@Cou@ZIF-8. After incubation, the cells incubated with Di-PEG@Cou@ZIF-8 showed higher fluorescence intensity than those with free Cou6 and mPEG@Cou@ZIF, as confirmed by confocal laser scanning microscopy (CLSM). Furthermore, LnCap cells were also incubated with free PTX, mPEG@PTX@ZIF-8, and Di-PEG@PTX@ZIF-8 to confirm their therapeutic effects. The Half-maximal inhibitory concentration (IC_50_) for free PTX, mPEG@PTX@ZIF-8, and Di-PEG@PTX@ZIF-8 after incubation for 48 h were 9.190, 0.1684, and 0.1052 μg/mL, respectively. Through the bivalent effect, Dipeptides significantly enhance cancer targeting in nanomedicine, and ZIF-8 facilitates the controlled rug release by responding to the pH of TME. These systems can effectively decrease non-specific accumulation and drug release, thus minimizing side effects.

#### 2.1.2. Cell Penetrating Peptide

**Figure 1:**
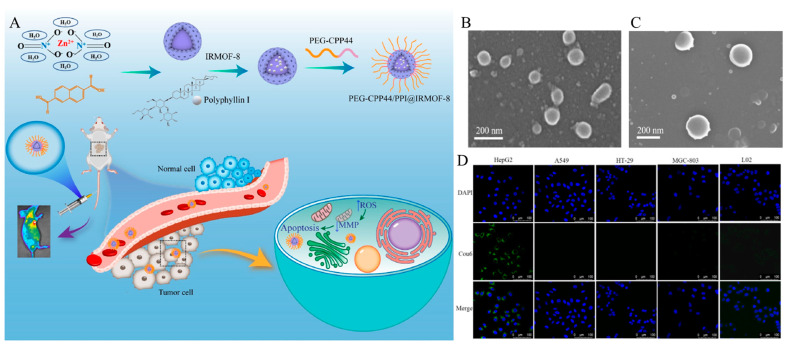
(**A**) Schematic illustration of the construction of functional metal-organic framework NPs and tumor therapy. (**B**) Scanning electron microscope image of IRMOF-8 and (**C**) PEG-CPP44/PPI@IRMOF−8. (**D**) CLSM image of different cells (HepG2, A549, HT−29, MGC−803, L0) after treatment of Cou6−labeled PEG−CPP44/IRMOF−8. Reproduced with permission [71]. Copyright (2023): Elsevier (Amsterdam, The Netherlands).

Cell-penetrating peptides (CPP) can transport hydrophilic macromolecules into cancer cells via an energy-independent pathway. Therefore, the penetration effect of the system can be increased through CPP modification. Wang et al. developed a cancer-targeting system using polyphyllin I (PPI), isoreticular metal-organic frameworks-8 (IRMOF-8), and cell-penetrating peptides (Figure 1A) [71]. PPI, naturally active steroid saponins, are effective for treating liver, lung, and stomach cancers. However, it has low solubility in aquatic solutions and damages normal cells and other side effects owing to its nonselective distribution [70]. IRMOF-8, which has a pore diameter close to the crystal size of the drug, was selected for encapsulation to achieve effective PPI delivery and has a diameter of 143.13 ± 7.42 nm (Figure 1B). In addition, it can be easily functionalized by hydrogen bonding with a polymer composed of PEG and a liver cancer-targeting peptide (CPP44, KRTPTMRFRYTWNPMK), and its diameter has increased to 202.97 ± 3.64 nm (Figure 1C). CPP44 can recognize liver cancer cells through high expression of M160. The thiol group at the end of the CPP44 structure and the maleimide at the end of mPEG2000 formed a thioether bond through a thiol-Michael addition click reaction. This polymer can be modified into a PPI-loaded MOF (PEG-CPP44/PPI@IRMOF-8), which increases the circulation time and cellular uptake of MOF. The Cou6 was labeled with a system for cellular uptake experiments. HepG2, L02, A549, MGC-803, and HT-29 cells were incubated with Cou6-labeled PEG-CPP44/PPI@IRMOF-8. After incubation, significant fluorescence was observed in HepG2 cells, but not in the L02, A549, MGC-803, or HT-29 cells (Figure 1D). This result suggests that PEG-CPP44/PPI@IRMOF-8 has selectivity for liver cancer cells. In the IRMOF-8-based system, modification of CPP33 endows the cancer-targeting effects, particularly selective targeting of liver cancer cells. In addition, IRMOF-8 was effectively loaded with PPI, which can treat liver cancer and lung cancer, increasing the drug stability and cancer treatment effect.

**Figure 2:**
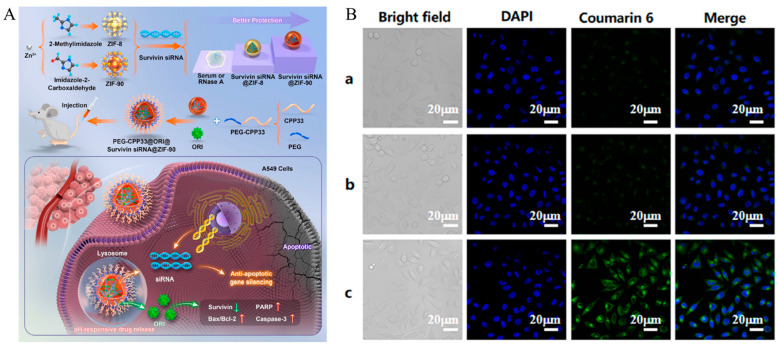
(**A**) Schematic illustration of the siRNA-based protection of ZIF-8 and ZIF-90 and anti-tumor mechanism of PEG-CPP33@ORI@Survivin siRNA@ZIF-90. (**B**) CLSM images for confirmation of uptake of (**a**) Coumarin 6, (**b**) Coumarin 6@ZIF-90, and (**c**) PEG-CPP33@Coumarin 6@ZIF-90 in A549 cells. Reproduced with permission [73]. Copyright (2023): Elsevier.

Small interfering RNA (siRNAs) are widely used in cancer treatment. However, the poor bioavailability and stability of siRNAs are the main problems in using siRNA for cancer therapy [72]. Cai et al. developed a cell-penetrating-peptide-modified MOF nanoparticle for siRNA delivery (Figure 2A) [73]. In this study, ZIF-90 was chosen as the drug delivery carrier. As ZIF-90 introduces an aldehyde group based on ZIF-8, it is more hydrophilic than ZIF-8, which can protect the loaded siRNA more effectively. Survivin siRNA and Oridonin (ORI) were loaded on ZIF-90 pores (ORI@survivin siRNA@ZIF-90) via electrostatic and π–π interactions. Survivin is a tumor-specific gene that is highly expressed and enhances apoptosis resistance in lung cancer cells [74]. Therefore, surviving siRNAs that inhibit survivin can effectively treat cancer if they co-deliver ORI, which causes cancer cell apoptosis [75]. After synthesis, PEG-drafted CPP33 (RLWMRWYSPRTRAYG), which specifically penetrates non-small-cell lung cancer A549 cells, was modified with drug-loaded ZIF-90 to obtain PEG-CP33@ORI@survivin siRNA@ZIF-90. The thiol group at the end of the CPP33 structure and the maleimide at the end of mPEG formed a thioether bond through a thiol-Michael addition click reaction. Coou6 was loaded instead of ORI or siRNA to confirm the cellular uptake of the system. A549, HepG2, HT-29, and MDA-MB-231 cells were incubated with Cou6, Cou6@ZIF-90, and PEG-CPP33@Cou6@ZIF-90. After incubation, strong fluorescence was observed in A539 cells incubated with PEG-CPP33@Cou6@ZIF-90, as confirmed by CLSM (Figure 2B). Saline, siRNA@ZIF-90, ORI@ZIF-90, ORI@survivin siRNA@ZIF-90 and PEG-CPP33@ORI@survivin siRNA@ZIF-90 was injected in A549 tumor-bearing nude mice. The tumor size in the PEG-CPP33@ORI@survivin siRNA@ZIF-90 treatment group was substantially smaller than that in other groups due to the combined effect of siRNA and ORI. Modified CPP33 imparts lung cancer targeting capabilities to a system based on ZIF-90. ZIF-90, being more hydrophilic than ZIF-8, encapsulates hydrophilic siRNA and exhibits better protective effects due to steric hindrance. This system effectively treated cancer cells by combining survivin siRNA and Oridonin. Cancer-targeting peptide-modified systems are summarized in Table 1.

### 2.2. Building Block of Self-Assembly Structure

In addition to using peptides as ligands to modify nanoparticles, they can also be utilized as drug-delivery vehicles by constructing self-assembled structures. Owing to their diverse physicochemical properties, peptides can form various nanostructured nanoparticles, nanotubes, nanofibers, and hydrogels with advantageous properties compared to conventional non-biological materials [82,83,84]. This unique morphology of the self-assembled structure can be controlled by modifying the self-assembly parameters, such as solvent, ionic strength, concentration, pH, temperature, and noncovalent interactions between the building blocks [85]. Self-assembled peptides not only have unique characteristics of peptides, such as biocompatibility and cancer-targeting properties but can also effectively deliver drugs by loading various drugs into their structure [86,87,88]. In addition, self-assembled peptide structures can be used to treat cancer cells [89].

#### 2.2.1. Drug Delivery Carrier

**Figure 3:**
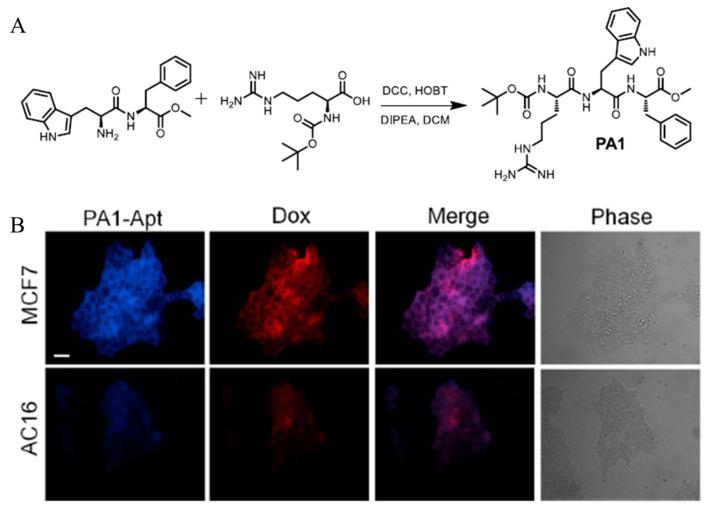
(**A**) Synthetic Methodologies Adopted for the Synthesis of PA1. (**B**) CLSM image of MCF7 and AC16 cells after incubation of PA1–Apt. Reproduced with permission [90]. Copyright (2023): American Chemical Society (Washington, DC, USA).

Once the peptide building blocks form a self-assembled structure, anticancer drugs can be loaded into the hydrophobic portion of the structure. Sivagnanam et al. utilized a self-assembled peptide structure as a drug delivery vehicle for cancer therapy [90]. The cationic tripeptide Boc–Arg–Trp–Phe–OME (PA1) self-assembles into spheres in aqueous solution. Each peptide sequence consisting of a PA1 peptide has a specific characteristic. The cationic amino acid Arg, which endows a net positive surface charge, strongly interacts with negatively charged cellular membranes and enhances the cellular uptake of the self-assembled structure. Trp has the highest emission maxima and a high quantum yield among aromatic amino acids, which endows the structure with fluorescent properties. The aromatic peptides, Trp and Phe, facilitate the self-assembly process by providing the π–π stacking interactions. Additionally, these aromatic peptides form a hydrophobic region in the self-assembled structure for drug loading. The critical aggregation concentration of PA1 was 0.962 mg/mL. An epithelial cell adhesion molecule (EPCAM)-directed aptamer was modified into PA1 to achieve a targeting effect [91]. The carboxylic acid group of the aptamers and the amine group of PA1 form an amide bond through the EDC reaction (Figure 3A). Self-assembly of the aptamer-modified PA1 was initiated in a solution containing doxorubicin (Dox) to form a Dox-loaded peptide structure (PA1–Apt). To confirm its cancer-targeting effects, human cardiomyocyte cells AC16 (EPCAM-negative) and human breast carcinoma epithelial cells MCF7 (EPCAM-positive) were incubated with PA1–Apt. Higher fluorescence intensities of the PA1-based assembly and Dox were observed in the MCF7 cells than in the AC16 cells (Figure 3B). These results indicated that PA1-Apt effectively targeted cancer cells. PA1 tripeptide in aqueous solution forms a spherical self-assembly structure. The hydrophobic interior of this structure allows for loading hydrophobic cancer drugs, overcoming low in vivo stability. The Arg amino acid present in the peptide imparts a positive charge to the system, aiding cell internalization with modified aptamers. This system facilitates selective targeting and treatment of breast cancer cells.

Sun et al. fabricated the self-assembled peptide structure using the diphenylalanine peptide (FF) derivative for cancer therapy [92]. 4,5-dihydroxyanthraquinone-2-carboxylic acid (Rhein), an anti-inflammatory compound, was combined with FF to improve π–π stacking and flexibility of the building block. Rhin-modified FF (RDP) forms a spherical structure through self-assembly and can be loaded with hydrophobic drugs.

To identify suitable compounds that can be effectively delivered by these nanoassemblies, structure-based virtual screening of a small-molecule library was performed, and camptothecine (CPT), which binds most strongly to RDP, was selected. π–π stacking and hydrophobic interaction between RDP and CPT were observed in virtual screening, and this effect strengthens the bonding. To observe the biodistribution of RDP nanoassemblies, the fluorescent substance 1,1′-dioctadecyl-3,3,3′,3′-tetramethylindotricarbocyanine iodide (DiR) and DiR-labeled RDP nanoassemblies were injected in 4T1 tumor-bearing Balb/c female mice. The DiR-labeled RDP exhibited higher fluorescence than free DiR in tumors. Furthermore, the tumor-inhibitory effects of the CPT-encapsulated RDP nanoassemblies and free CPT were compared in 4T1 xenograft tumor-bearing mice. Within the observation period of 11 days, CPT-encapsulated nanoassemblies significantly inhibited tumor growth compared with free CPT. FF dipeptide derivatives form self-assembly structures and load hydrophobic CPT. This system can effectively deliver water-insoluble drugs to cancer cells and increase treatment effectiveness.

Cong et al. developed a transformable nanosystem based on peptide self-assembly [93]. Thermodynamically stable telodendrimers, which are self-assembled building blocks, were synthesized by modifying fluorophenylboronic acid (PBA), an indocyanine green derivative (ICGD), and cholic acid (CA) into an octapeptide chain (KEKEKE). CA and ICGD form a hydrophobic core in the structure, allowing the loading of the cancer drug 7-ethyl-10 hydroxycamptothecin (SN38), and the hydrophilic shell has a positive charge. Peptide-based self-assembled nanoparticles (ICP NP) have a 23.8 nm hydrodynamic particle and a critical micelle concentration for the self-assembly of 0.04 μM. For ICP NP surface modification, negatively charged hyaluronic acid (HA) functionalized with dopamine and folic acid was prepared. Electrostatic interactions between HA and ICP building blocks, borate binding by dopamine present in HA, and PBA present in the ICP building blocks promoted the formation of HA-modified ICP NPs (hICP NPs). The size of the hICP NPs was 130 nm, which facilitated tumor accumulation through the EPR effect. The hICP NPs, which accumulate in the tumor tissue, are decomposed by the low pH and high HAase concentration in the tumor microenvironment, transforming into ICP NPs and improving tumor penetration. Additionally, positively charged ICP NPs interact with negatively charged cell membranes, promoting the cellular uptake of NPs and resulting in effective SN38 delivery. The cellular uptake and tumor penetration effects of these transformable NPs were confirmed in B16 melanoma cells and B16 tumor cell spheroids. When free ICGD, ICP NP, and hICP NP were incubated with B16 melanoma cells, strong fluorescence was observed in the group incubated with ICP NP and hICP. Additionally, when ICP and hICP NPs were incubated in B16 melanoma tumor cell spheroids, ICP NP and pre-incubated hICP NP at pH 6.0 and the 60 mU/mL HAase group showed improved tumor penetration. In contrast, tumor penetration was delayed in the group in which hICP NPs were incubated directly without free incubation. This implied that hICP NPs were transformed by low pH and HAase to form ICP NPs and promote tumor penetration. Peptide-based telodendrimers form transformable self-assembly structures through interaction with HA. These systems, which respond to pH and enzymes, change their size and surface charge to enhance deep tissue penetration and cellular uptake of the encapsulated drug.

#### 2.2.2. Therapeutic Peptide

The self-assembled peptide structure is used not only as a drug carrier but also to treat cancer directly. Zhou et al. developed a peptide sequence using structure-based virtual screening techniques [94]. In this study, a self-assembling D-peptide supramolecular nanomedicine (NMTP-5) targeting neuropilin-1 (NRP1) and mouse double-minute 2 (MDM2) was developed using structure-based virtual screening techniques. MDM2 is a negative regulator of p53 and transactivates the expression of target genes that mediate cell cycle arrest and apoptosis. MDM2 is overexpressed in various cancers, promotes p53 degradation, and inhibits p53 protein levels by binding to p53 [95]. Therefore, upregulating p53 levels by interfering with the MDM2–p53 interaction can effectively treat cancer cells. NRP1 is a transmembrane glycoprotein that is overexpressed in cancer cell membranes and regulates the cell penetration ability of peptide drugs. Therefore, NRP1-targeting peptide can effectively penetrate deep into tumors to treat cancers [96]. The sequence of NMTP-5 was ZFFYGWYGGMEKLLRGGRGERPPR, and the ZFFY sequence allowed the self-assembly of the peptide. The critical aggregation concentration of NMTP-5 is 36.2 μm and forms the fiber shape. To confirm cellular uptake, isothiocyanate-loaded NMTP-5 was incubated in SK-Hep-1 cells and SK-Hep-1 cells in which NRP1 was silenced by NRP1-targeting shRNA. Strong fluorescence was observed in SK-Hep-1 cells, whereas weak fluorescence due to NMTP-5 uptake was observed in NRP1-silenced cells. To investigate whether NMTP-5 inhibited the binding of endogenous MDM2 to p53 in SK-Hep-1 cells, an immunoprecipitation assay was performed. NMTP-5 inhibits the interaction between endogenous MDM2 and p53 and increases the p53 protein level in SK-Hep-1 cells. Moreover, injecting NMTP-5 into a Sk-Hep-1 cell-derived xenograft model effectively suppressed tumor growth. Structure-based virtual screening techniques were used to select peptides targeting NRP1 and MDM2, leading to the development of NMTP-5. These peptides not only serve as building blocks for self-assembly but also enable cancer targeting and therapy.

**Figure 4:**
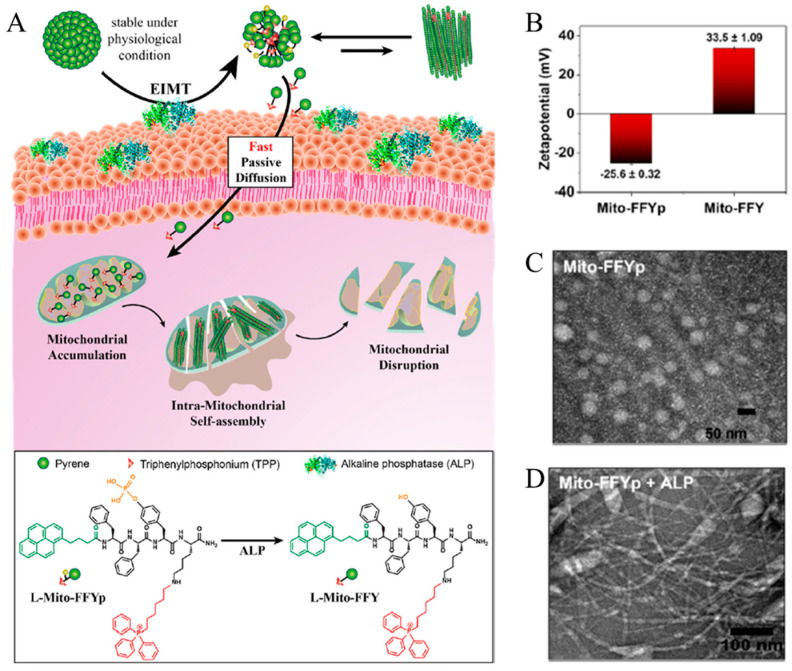
(**A**) Schematic illustration of the enzyme-instructed morphology transformation (EIMT) of an ALP-responsive micelle. The ALP enzyme induces the transformation of Mito−FFYpK micelles to positively charged Mito-FFYK molecules, which enter the cell via free diffusion to target the mitochondria. Inside the mitochondria, Mito−FFYpK self-assembles into nanofibers to induce mitochondrial dysfunction leading to cell death. The chemical structure of Mito−FFYpK and Mito−FFYK is represented under the scheme. (**B**) Surface charge analysis of Mito−FFYpK and Mito-FFYK showing negative potential for Mito−FFYpK micelle and positive potential for Mito−FFYK. TEM analysis of 15 mM (**C**) L−Mito−FFYpK and (**D**) L−Mito−FFYpK + ALP. Reproduced with permission from Reference [97] Copyright (2022): The Royal Society of Chemistry.

In addition, Jeena et al. developed a self-assembly building block by modifying the peptide backbone with pyrene, and triphenylphosphonium (TPP) ligand targeting mitochondria (Figure 4A) [97]. FFYpK peptide was synthesized by adding L-tyrosine phosphate unit (Yp) and lysin to dipeptide (FF). The phosphate unit present in the peptide sequence is cleaved by the alkaline phosphatase (ALP) enzyme, which is overexpressed in cancer cells, to form FFYK [98]. FFYpK peptide N terminus forms an amide bond with the carboxylic acid present in pyrene, and the amine group present in the lysine side chain forms a secondary amine with 1-hexyl triphenylphosphonium bromide. FFYpK (Mito-FFYpK) modified with pyrene and TPP has a micellar structure and a negative surface charge. On the contrary, Mito-FFYK, in which the phosphate unit has been removed by the ALP enzyme, has a fiber-shaped structure and a positive surface charge (Figure 4B). Therefore, when the micellar structure of Mito-FFYpK reaches cancer cells, it is converted to Mito-FFYK by ALP present in the cancer cell, and since the building block is positive, it easily internalizes the cancer cell membrane and selectively accumulates in the mitochondria (Figure 4C,D). When the concentration of Mito-FFYK accumulated in mitochondria exceeds the critical aggregation concentration (15 μM), it forms a fibrous structure, causing mitochondrial dysfunction and inducing the death of cancer cells. To confirm the cancer therapy effect, Mito-FFYpK was incubated in Saos-2, HeLa, SK-BR-3, and NIH-3T3 cell lines. The viability of SK-BR-3, and NIH-3T3 cell lines, which weakly express ALP enzymes, was 90% at 10 µM Mito-FFYpK. Meanwhile, Saos-2 and HeLa show 50% cell viability at about 5 µM Mito-FFYpK due to the high ALP expression. This suggests that the cytotoxicity of Mito-FFYpK is selective toward the ALP enzyme expression. The peptide derivative that responds to ALP increases the selective treatment effect of cancer cells through intramitochondrial self-assembly and is a system that effectively treats cancer by inducing dysfunction of the mitochondria of cancer cells. Self-assembled peptide-modified systems have been summarized in Table 2.

### 2.3. Stimuli-Responsive System

The premature leakage of drugs from nanocarriers during delivery to cancer cells is a major problem, leading to nonspecific drug distribution and the potential harm or death of normal cells [104,105]. This challenge can be overcome by modifying cancer-targeting ligands; however, it can also be addressed by controlling the encapsulated drug release [23,106,107]. The use of stimuli-responsive peptides in nanocarriers facilitates controlled drug release [108]. This approach not only minimizes damage to healthy cells but also increases the effectiveness of cancer therapy by ensuring that the drug is released at the site of the tumor in a controlled manner.

#### 2.3.1. Enzyme Responsive Peptide

**Figure 5:**
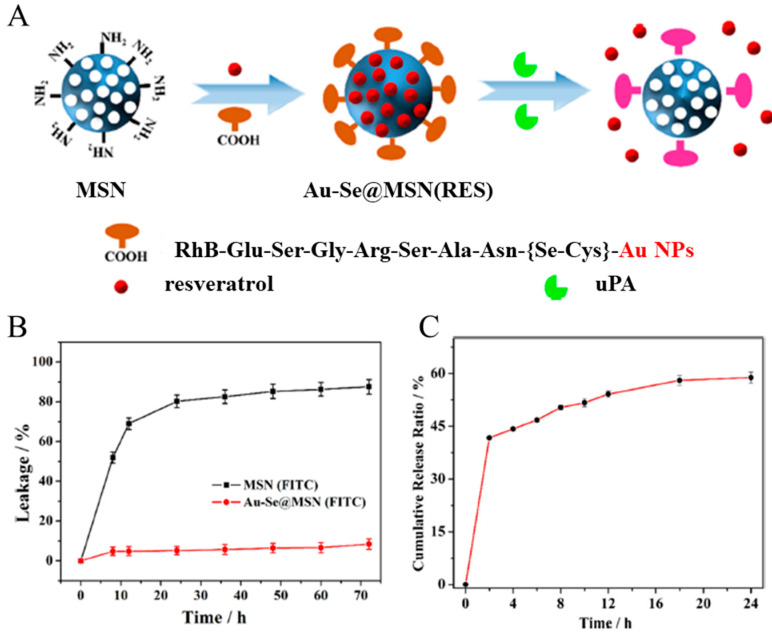
(**A**) Design of the Au–Se@MSN (resveratrol). (**B**) Drug leakage of Au–Se@MSN(FITC) and MSN(FITC). (**C**) uPA-stimulated drug release profile of Au–Se@MSN (resveratrol). Reproduced with permission from Reference [109] Copyright (2023): American Chemical Society.

Peptides that are degraded or activated by specific enzymes react depending on the presence or absence of the enzyme. Therefore, a system can be developed to release drugs using peptides that react with enzymes overexpressed in cancer. Liu et al. developed a system that controls drug release by blocking the pores of MSNPs with GNPs using a ligand containing a peptide sequence (Figure 5A [109]). A peptide substrate containing a urokinase-type plasminogen activator (uPA)-specific responsive peptide sequence (ESGRSAN), glutamic acid, and selenocysteine termini was labeled with rhodamine B [110]. This peptide substrate forms a gold selenium linkage (Au–Se) with the GNP and an amide bond with the silica nanoparticles, thereby forming a GNP-modified MSN (Au–Se@MSN). Additionally, the uPA-specific peptide constituting the ligand is cleaved by uPA, which is highly expressed in metastatic cancers. This phenomenon detaches GNPs capping the MSN pores and facilitates cancer-specific drug release. To confirm the leakage and responsive release of the cargo from Au–Se@MSNs, a release assay was conducted by loading isothiocyanate (FITC) into Au–Se@MSNs and MSN. Although 80% of FITC was released from the MSN group, the Au–Se@MSN group exhibited insignificant FITC release (Figure 5B). After being simulated with 0.4 μg/mL uPA, the FITC cumulative release rate of the Au–Se@MSN group reached up to 60% (Figure 5C). This suggests that the peptide sequence effectively controls the drug release via enzymatic stimuli. Au–Se@MSN loaded with resveratrol significantly inhibited tumor growth in an in vivo experiment. Se-Cys containing peptides, modified with nanomedicine through Au-Se bonds with GNP, block pores to prevent drug leakage and respond to uPA for selective drug release in cancer. This system can effectively reduce drug side effects and increase cancer treatment.

As mentioned above, peptides endow multi-functionality to nanomedicine. In peptide-based self-assembled nanomedicine, the peptide acts as a building block and causes the formation or collapse of the self-assembled structure in response to stimuli. Yang et al. developed a self-assembled structure comprising a Dox prodrug [111]. The Dox prodrug was constructed by forming an amide bond between the cathepsin B-cleavable peptide (FRRG) and Dox. This direct FRRG conjugation to Dox prevented premature drug release in normal tissues. Through hydrophobic interactions and π–π stacking, peptide-modified prodrugs self-assemble into cancer-activating Dox prodrug nanoparticles (CAP-NPs) that are specifically degraded into cytotoxic Dox molecules by cathepsin B, an enzyme overexpressed in cancer cells. To compare the cancer therapeutic effects, saline, free Dox, and CAP-NPs were injected into CT26 tumor-bearing mice. CAP-NP-treated mice showed minimal tumor growth compared with the saline- and free Dox-treated mice. A prodrug composed of peptide and DOX reacts with an enzyme to selectively release the drug from cancer cells. This system prevents damage to normal cells and targets cancer cells selectively, increasing cancer treatment efficacy.

**Figure 6:**
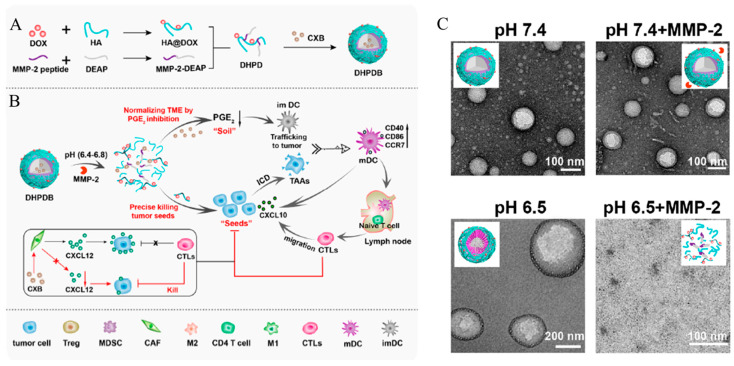
(**A**) Schematic illustration of the detachable nanoparticles for cancer immunotherapy synthesis of the detachable vesicles. (**B**) Schematic depiction of the anti-tumor immunotherapy elicited by vesicles in vivo. (**C**) TEM images of DHPD. Reproduced with permission from Reference [112] Copyright (2020): American Chemical Society.

Wang et al. developed a self-assembled structure comprising a pH-responsive molecule and HA-grafted Dox (Figure 6A) [112]. The pH-responsive molecule 3-diethylaminopropyl isothiocyanate (DEAP) and HA-grafted Dox were conjugated to a peptide (GRVGLPG) cleavable by MMP-2, which was overexpressed in cancer cells (Figure 6B). The N-terminus of the peptide forms thiourea with the thiocyanate of DEAP, and the carboxylic acid at the C-terminus and HA forms two amide bonds with diethyl amine. The building blocks were DEAP, HA-grafted Dox, and peptide-formed spherical nanoparticles (DHPD). DEAP constitutes the hydrophobic core of the micelles, enabling hydrophobic drug loading. It is protonated at tumor pH and promotes changes in the self-assembled structure (Figure 6C). COX-2 inhibitor celecoxib (CXB)-loaded nanoparticles (DHPDB) were constructed using a self-assembly method [113]. As high COX-2 expression interferes with T cell anti-tumor effects in several ways, including blocking dendritic cell (DC) migration, the combination of CXB and Dox has a synergistic effect on the effectiveness of cancer treatment. The peptide present in DHPDB is cleaved by MMP-2, which is overexpressed in cancer, and the self-assembled structure is decomposed due to DEAP protonation, thereby releasing CXB. Furthermore, Dox grafted onto HA was released by HAase and increased the efficacy of cancer therapy. To confirm the effect of cancer therapy, free Dox, free CXB, DHPD, and DHPDB were injected into 4T1 tumor-bearing BALB/c mice. DHPDB showed the highest tumor growth inhibition effect. The self-assembled structure, which contains a peptide that is degraded by MMP-2, selectively releases the loaded drug from cancer cells and activates the immune system. CXB and Dox show a synergy effect and facilitate more effective immunotherapy.

Wu et al. overcame cancer hypoxia using a nanocomposite (RKCM) consisting of a peptide, chlorine e6 (Ce6), and MnO_2_ [114]. The self-assembled structure of RKCM was constructed by a peptide-drafted naphthalene through an amide bond with three functional segments, Nap-FFGPLGLARKRK (abbreviated as RK). In the first segment, the Nap-FF- segments endowed hydrophobicity and facilitated the hydrophobic Ce6. The second segment, GPLGLA, is cleaved by metalloproteinase-7 (MMP-7) to facilitate the stimuli-responsive drug release. The third segment, –RKRK, provides a positive charge to form a nanofiber structure. RK can self-assemble into a fibrillar structure, and MnO_2_ can be mineralized on its surface to obtain RKCM. The RKCM nanocomposite can overcome hypoxia in tumor tissues because the coated MnO_2_ can supply O_2_ by reacting with H_2_O_2_ [115]. Ce6 induces ^1^O_2_ generation for photodynamic therapy (PDT) [116]. Furthermore, RKCM achieved tumor-targeted delivery in response to tumors expressing MMP-7. To confirm the effects of cancer therapy, free Ce6 and RKCM were injected into 4T1-tumor-bearing mice. The RKCM group showed higher tumor growth inhibition than the free Ce6 group in the presence of laser irradiation. This suggests that RKCM delivers Ce6 to cancer cells and increases the PDT effect by generating O_2_. The RK peptide, which consists of a hydrophobic, enzyme-sensitive, and positively charged segment, forms self-assembled fibrils, which react with MMP-7 to release the loaded photosensitizer from cancer cells, enabling PDT under light irradiation. This dual stimuli-responsive system enhances cancer targeting and increases the cancer therapy’s effect.

#### 2.3.2. pH-Responsive Peptide

The natural amino acids lysine (K), arginine (R), histidine (H), and glutamic acid (E), which contain ionizable groups such as carboxyl, imidazole, primary amine, and tertiary amine, respond to pH by changing the charge of the molecule. Therefore, by introducing amino acids with ionizable groups into the peptide sequence, a system that responds to specific pH levels can be developed. Wang et al. studied a system that responds to the TME pH using a peptide-rich in H and K (KKKHHHH-Acp-LLLLLLLLGSPDRGD, where Acp stands for 6-aminocaproic acid) [117]. At pH 7.4, the peptide forms a self-assembled structure through electrostatic and hydrophobic interactions, capable of loading nucleic acid. The K sequence of the peptide becomes protonated at TME pH, gaining a positive charge, which, along with the peptide’s RGD, enhances the uptake of nucleic acid into cancer cells. The H sequence of the peptide is protonated in lysosomes, which have a lower pH, increasing the peptide’s positive charge. This increase in positive charge enhances the electrostatic repulsion between the peptides, triggering the release of the encapsulated nucleic acid drug. This multi-pH responsive system enables not only targeted cancer therapy but also controlled drug release.

#### 2.3.3. Light Responsive Peptide

Peptides that respond to light can be synthesized by modifying the photoreactive protecting group on the peptide. PG reactivates the charge of the peptide via NIR two-photon photolysis, allowing rapid penetration into cancer cell membranes. Yang et al. developed a system based on a photo-sensitive peptide (PSP) modified liposome, which targets the cancer cell in response to light stimuli [118]. The sequence of the CPP is CGRRMKWKK, which does not possess inherent cancer-targeting ability. PSP is formed by a covalent bond between PG (1-(bromomethyl)-4,5-dimethoxy-2-nitrobenzene) and the amine group of the lysine side chain of CPP via an S_N_2 reaction. Additionally, PSP is modified onto the liposome, which is composed of 1,2-Distearoyl-sn-glycero-3-phosphoethanolamine-N-methoxy(polyethyleneglycol) (DSPE-mPEG2000), through a thiol-Michael addition click reaction. The PSP-modified liposome (PSP-L) can act as a carrier for vinorelbine bitartrate (VB). Moreover, PSP-L can target cancer via the EPR effect. Upon reaching cancer cells and under NIR irradiation, PG undergoes photodegradation, exposing the CPP moiety and increasing the cancer penetration of liposomes. This system utilizes PSP, which responds to light stimulation, to enable cancer targeting, which is lacking in existing CPP. The stimuli-responsive peptide-modified systems are summarized in Table 3.

## 3. Conclusions

This review comprehensively explores the innovative integration of peptide functionalization within the realm of nanomedicine for advancing cancer therapy. Peptides are ideal candidates for enhancing the efficacy of nanocarriers in targeting cancer cells owing to their unique properties, including specificity, biocompatibility, and versatility. The primary focus of this study was on the three pivotal roles of peptides as cancer-targeting ligands, building blocks for self-assembling nanostructures, and integral components of stimuli-responsive systems.

Nanoparticle modification with cancer-targeting peptides represents an important step in oncological treatments. These peptides not only facilitate the selective delivery of therapeutic agents to cancer cells but also enhance cell membrane penetration, ensuring more effective drug uptake directly into cancer cells. This dual functionality reduces the adverse effects on healthy tissues and improves delivery efficiency. This targeted approach not only enhances the therapeutic index of conventional treatments but also addresses the challenges of non-specific drug distribution and systemic toxicity, offering a precise and effective method for cancer treatment.

Second, peptides are used as building blocks for self-assembling nanostructures. This application of peptides is particularly noteworthy, as it may create diverse nanoarchitectures such as nanoparticles, nanotubes, and hydrogels. These structures, formed through self-assembly, can not only encapsulate therapeutic agents and offer a controlled and sustained release mechanism but also disrupt cellular organelles, providing an innovative approach to cancer therapy. Targeting and disrupting specific cellular structures within cancer cells is a unique and effective method for combating cancer. The inherent biocompatibility of peptides further enhances the appeal of these nanostructures, making them suitable for in vivo applications, and offering a promising avenue for advanced cancer therapies.

Finally, the development of peptide-based stimuli-responsive systems has considerably improved targeted therapies. These systems are designed to respond to specific stimuli within the tumor microenvironment, such as pH changes or the presence of enzymes, which trigger the release of encapsulated drugs directly at the tumor site. This precise drug release not only maximizes the therapeutic effect but also minimizes collateral damage to the surrounding healthy tissues.

In conclusion, the integration of peptide functionalization into nanomedicine represents a promising frontier in cancer therapy. The versatility of peptides in enhancing targeting specificity, enabling complex nanostructure construction, and facilitating stimuli-responsive drug release pave the way for effective and safe cancer treatments.

## Figures and Tables

**Table 1:** Summary of cancer-targeting peptide modified systems.

Nanoparticle	Peptide Sequence	Functionalization Method	Drug	Cancer Type	Ref.
GNP	WXEAAYQRFL	Host–guest interaction	Plasmid DNA	Breast cancer	[61]
ZIF-8	WQPDTAHHWATL	Electrostatic interaction	PTX	Prostate cancer	[63]
GNP	CRGDK	Amide bond	TSFAEYWNLLSP	Breast cancer	[76]
GNP	RGDyC	Au-thiol bond	N/A	Cervical cancer	[77]
GNP	KTLLPTP	Au-thiol bond	Gemcitabine	Pancreatic cancer	[78]
MSN	RGD	Amidebond	PTX	Breast cancer	[79]
IRMOF-8	KRTPTMRFRYTWNPMK	Hydrogen bonding	PPI	Liver cancer	[71]
ZIF-90	RLWMRWYSPRTRAYG	Electrostatic interaction	siRNA, ORI	Lung cancer	[73]
MSN	cCPGPEGAGCLLIILRRRIRKQAHAHSK	Amidebond	Epigallocatechin-3-gallate	Breast cancer	[80]
MSN	CGRKKRRQRRRPPQRGDS	Disulfide	Dox	Cervical cancer	[81]

**Table 2:** Summary of self-assembly peptide modified systems.

Structure Morphology	Peptide Sequence	Self-Assembly Driving Force	Drug	Cancer Type	Ref.
Sphere	RWF	π–π stacking interactions	Dox	Breast cancer	[90]
Sphere	FF	π–π stacking interactions	CPT	Breast cancer	[92]
Sphere	KEKEKEKE	Hydrophobic interaction	SN38	Skin cancer	[93]
Fiber	KKAAAAAAK	Hydrophobic Interaction Hydrogen bonding	PTX	Breast cancer	[99]
Sphere	W	π–π stacking interactions	Dox	Glioma	[100]
Sphere	KFG	π–π stacking interactions Hydrogen bonding	Dox	Cervical cancer	[101]
Fiber	ZFFYGWYGGMEKLLRGGRGERPPR	π–π stacking interactions	Self-assembly structure	Liver cancer	[94]
Fiber	FFYK	π–π stacking interactions	Self-assembly structure	Osteosarcoma Cervical cancer	[97]
Sphere	ffkkfklklk	π–π stacking interactions	Self-assembly structure	Cervical cancer	[102]
Fiber	KLVFF	π–π stacking interactions	Self-assembly structure	Lung cancer Cervical cancer	[103]

**Table 3:** Summary of stimuli-responsive peptide modified systems.

Nanoparticle	Peptide Sequence	Stimuli	Functionalization Method	Drug	Cancer Type	Ref.
MSN, GNP	ESGRSAN	uPA	Amide bond, Au-Se linkage	Resveratrol	Breast cancer	[109]
Self-assembly structure	FRRG	Cathepsin B	Amide bond	Dox	Colon cancer	[111]
Self-assembly structure	GRVGLPG	MMP-2	Amide bond, thiourea bond	Dox, CXB	Lung cancer	[112]
Self-assembly structure	FFGPLGLARKRK	MMP-7	Amide bond	Ce6	Breast cancer	[114]
Self-assembly structure	FRRG	Cathepsin B	π–π stacking hydrophobic interaction	Dox	Colon cancer	[119]
Self-assembly structure	DEVD	Caspase-3	π–π stacking hydrophobic interaction	Dox	Colon cancer	[120]
Self-assembly structure	KKKHHHH-ACP-LLLLLLLLGSPDRGD	pH	Electrostatic interaction Hydrophobic interaction	Nucleic acid	Cervical cancer	[117]
Self-assembly structure	FFKK	pH	Electrostatic interaction	Self-assembly structure	Cervical cancer	[121]
Self-assembly structure	D, βA	pH	π–π stacking	Self-assembly structure	Breast cancer	[122]
Liposome	CGRRMKWKK	Light	Thiol-Michael addition	VB	Breast cancer	[118]
